# Life at Both Ends of the Ladder: Education-Based Identification and Its Association With Well-Being and Social Attitudes

**DOI:** 10.1177/0146167215594122

**Published:** 2015-09

**Authors:** Toon Kuppens, Matthew J. Easterbrook, Russell Spears, Antony S. R. Manstead

**Affiliations:** 1 University of Groningen, The Netherlands; 2 University of Sussex, Brighton, UK; 3 Cardiff University, UK

**Keywords:** education, identification, well-being, political attitudes, prejudice

## Abstract

Level of formal education is an important divide in contemporary societies; it is positively related to health, well-being, and social attitudes such as tolerance for minorities and interest in politics. We investigated whether education-based identification is a common underlying factor of these education effects. Indeed, education-based identification was stronger among the higher educated, especially for identification aspects that encompass education-based group esteem (i.e., the belief that one’s educational group is worthy and that others think so, too). Furthermore, while group esteem had beneficial effects across educational levels, aspects of identification that were unrelated to group esteem had positive effects for the higher educated but not for the less educated. Thus, the less educated do not benefit from the psychologically nourishing effect of identification that exists for other groups. The stigma and responsibility related to low education could be a common explanation for a wide range of outcomes.

Social psychologists have devoted much attention to groups based on gender, race, ethnicity, and age. Although level of formal education is also one of the major divides in contemporary societies, there has been almost no research that has specifically investigated psychological processes among education-based groups. This is surprising given that low levels of formal education are related to poor health, lower life expectancy, and lower personal well-being (Marmot & Wilkinson, 2006), as well as social attitudes that threaten social cohesion and engagement, such as prejudice, lack of interest in politics, and lack of trust in others (Elchardus & De Keere, 2013). We refer to the association between education and all these outcomes collectively as the *education effect*, and in the present research, we aim to understand the psychological bases of this effect.

Although the existence of the education effect clearly implies that education is an important topic for investigation, research on education as a predictor of well-being and societal attitudes has thus far been mainly rooted in sociology. This is despite the potential for proximal psychological processes to provide a common underlying explanation for the diverse outcomes that are included in the education effect. In the current research, we argue that those with lower levels of education have difficulty in constructing a positive social identity around their level of education and that this common cause can to a large extent explain the education effect.

## The Importance of Education

Empirical approaches to “social class” range from occupation-based class systems inspired by sociological theory (see Crompton, 2008) to measures in which participants are asked to place themselves relative to others in terms of income, education, and occupational status (Kraus, Piff, Mendoza-Denton, Rheinschmidt, & Keltner, 2012). Here, we focus more narrowly on education for two reasons. First, a low level of education is related to a wide range of outcomes, over and above the effects of other aspects of socioeconomic status (Easterbrook, Kuppens, & Manstead, 2015). Second, education has become increasingly important both personally and societally; education is economically beneficial for individuals (Day & Newburger, 2002) and countries (Aghion, Boustan, Hoxby, & Vandenbussche, 2009), and its importance as a determinant of class position (Featherman & Hauser, 1976; Grusky & DiPrete, 1990) and choice of marriage partners (Hou & Myles, 2008) has increased over time.

This is consequential. Lower levels of education are related to poor health, higher rates of depression, and suicide (Hudson, 2005; Marmot & Wilkinson, 2006; Ritsher, Warner, Johnson, & Dohrenwend, 2001), as well as to societal attitudes such as increased prejudice (Pettigrew et al., 1997), exclusionist and authoritarian attitudes (Coenders & Scheepers, 2003), and less political engagement (Persson, 2013). Moreover, education often has a stronger relation with these outcomes than do variables such as income or occupation (e.g., Easterbrook et al., 2015; Smets & Van Ham, 2013). Thus, there are good reasons to study education effects. Below, we report three studies in which we include a range of outcomes that previous research has indicated are different manifestations of the education effect and offer a psychological perspective that is intended to explain commonalities across these different manifestations.

## Education and Social Identity

In terms of psychological processes, one relevant aspect of the increased importance of education as an indicator of social status is that it represents a change in the perception of social status from being primarily *ascribed* to being primarily *achieved*. Social inequalities have always existed, but with the increased importance of education, inequalities are now more easily perceived as being individuals’ personal responsibility (the ideology of “meritocracy”; Tannock, 2008). We argue that this has profound consequences for the way in which those with less education see themselves and the way they deal with their low-status position. While high status groups furnish their members with a positive social identity from which they can derive self-esteem, belonging to a low-status group amounts to having a negative social or collective identity that can be threatening to self-esteem (Tajfel & Turner, 1986). We propose that less educated people have difficulties in dealing with the negative identity that is associated with being less educated and that this is an underlying mechanism explaining many of the education effects (Ellemers, Spears, & Doosje, 2002).

How do those who have low status cope with their negative social identity? Some people react to their low status by forming a social identity around their status group and identifying with others who share their social position. Identifying with a social group has been consistently found to be positively related to health and well-being (Jones & Jetten, 2011; Sani, 2012) and to buffer against the negative consequences of perceived discrimination (Schmitt & Branscombe, 2002). Several mechanisms have been proposed to explain this link, including an increase in perceived social support from in-group members (Sani, 2012), a cognitive sense of resilience to and rejection of stigma (Crabtree, Haslam, Postmes, & Haslam, 2010), and a heightened sense of personal control (Greenaway et al., 2015). Thus, if the less educated can form a social identity and, therefore, identify with their educational group, their well-being is likely to be enhanced.

However, there are several factors that are known to discourage identification that are directly relevant to the case of the less educated, which may undermine the beneficial consequences of identification. First, because education is often perceived to be a legitimate way of according status to people (Bourdieu, 1984), less educated people might not perceive themselves to be the victims of discrimination. This renders their low status legitimate, which discourages group-based identification (Jetten, Schmitt, Branscombe, Garza, & Mewse, 2011). Second, education can also be seen as a system that rewards characteristics of individuals rather than groups, and this individual nature of group assignment also reduces the salience of group-based identities and, therefore, reduces identification (Ellemers, 1993). Third, being less educated is defined by an absence of something, that is, *a lack* of education. This is in contrast to other stigmatized identities where the group-defining element (e.g., being a woman) is often perceived to be multifaceted and to consist of many potentially positive elements, at least by those who are stigmatized. For these reasons, we question whether the less educated are able to benefit from the buffering effect of identification. This is the key empirical question of the present research.

Although our argument hitherto has focused mainly on well-being outcomes, we expect similar processes to account for the relationship between education level and social attitudes. For example, if low-status groups cannot construct a positive social identity, then group members may resort to denigrating outgroups of similar status in an attempt to raise the *relative* status of their in-group by lowering the status of a perceived competitor. Applying this to those with low levels of education could explain why they show heightened levels of racism and anti-immigrant attitudes. This is important because such attitudes have often been attributed literally and directly to a lack of education (Lipset, 1981), rather than, as we propose, to the stigmatic and esteem consequences of this identity.

Yet another reaction to being a member of a group whose low status is perceived to be legitimate is simply to withdraw from areas associated with the low-status attribute. Given the increased importance that society places on education, and the overrepresentation of the highly educated in politics, this could be reflected in the lack of political engagement reported by the lower educated.

## The Present Research

We predict that people with lower levels of education identify less with their educational group compared with more highly educated people. Surveys in Denmark and Flanders found that the less educated are less likely to respond to a forced-choice question in which they are asked to indicate whether they identify with higher educated or less educated people (Spruyt & Kuppens, 2014; Stubager, 2009). A limitation of those studies is that the identification measure used asked merely whether people identified with higher educated, less educated, or neither.

By contrast, our approach to measuring identification is more in line with psychological research on social identification. We first analyze people’s degree of identification with their own education level (rather than making them choose between two broad education levels) using a single-item measure that was included in two large, representative British surveys. We then conduct our own experimental studies in which we define people’s educational group and assess their identification with this particular group using a multidimensional identification scale (Leach et al., 2008). We examine whether there are differences in the effects and responses associated with the different facets of identification, which remain hidden in studies using single-item identification measures.

Our second aim is to investigate whether identification with one’s educational group plays a role in any or all of the outcome variables that are known to be related to education. Given the evidence for the beneficial effect of group memberships and identification (Iyer, Jetten, Tsivrikos, Postmes, & Haslam, 2009; Jetten, Haslam, & Haslam, 2012; Jones & Jetten, 2011), we could expect positive effects, regardless of education level. However, it is also possible that the negative social identity of the less educated precludes any positive effects of identification, or even leads to negative effects.

## Overview of Studies

In Study 1, we use two representative samples of the U.K. population to assess the relation between education and identification, providing evidence that people do use their level of education to define who they are, at least to the same extent that they use other, more frequently studied social categories. We also show that there are important differences in the degree to which education is incorporated into the self-concept depending upon one’s level of education. Study 2 replicates this effect and additionally shows that educational differences are strongest for *affective* identification or *group esteem*—the satisfaction people feel about belonging to a group. Studies 1 and 3 (with a U.S. sample) also explore the relation between identification and the outcome variables relevant to the education effect. Finally, Study 3 adds a manipulation of the salience of education, allowing us to investigate the impact of a cue indicating that education is important within this particular context. This is important because it allows us to go beyond correlational interpretations of the data and also because it mirrors the increase in the weight society attaches to education.

## Study 1

We use representative samples of the U.K. population to investigate (a) people’s identification with their educational group and whether this is related to their educational level and (b) the relation between identification and outcomes known to be related to education.

### Method

#### Datasets and samples

We identified two datasets that contained items that were appropriate for our purposes: the Understanding Society Study (USS) and the Citizenship Survey (CS).

##### USS

The USS is a longitudinal study of a representative sample of around 40,000 U.K. households. We analyzed data from Wave 2, which were collected via interviews and self-completion questionnaires in 2010-2011. We focused exclusively on those who were not currently involved in full-time education, which, with listwise deletion across the predictor and control variables, left 27,467 respondents (age range = 16-102 years, *M_age_* = 47.17, *SD_age_* = 16.66, 56% female). More information can be found on the USS website (https://www.understandingsociety.ac.uk).

##### CS

The CS is a (now discontinued) biannual survey of a regionally representative sample of around 15,000 adults in England and Wales. We analyzed data collected via interviews in 2010-2011. We again focused exclusively on respondents who were no longer in full-time education, which, with listwise deletion across the predictor and control variables, left 11,737 respondents (age range = 16-69 years, *M_age_* = 41.45, *SD_age_* = 14.09, 54% female). More information can be found on the CS website (http://webarchive.nationalarchives.gov.uk/20120919132719/www.communities.gov.uk/communities/research/citizenshipsurvey/).

#### Education

In both the USS and the CS, respondents’ highest educational qualification was categorized as follows: no qualifications, General Certificate of Secondary Education (GCSE) or equivalent, A-Level or equivalent, higher education qualification below degree level, degree or equivalent, and above (see Table 1 for frequencies). For our main analyses, we dichotomized respondents’ highest educational qualification into those who had achieved a university degree versus those who did not. We did this for several reasons. First, the education effect is mainly driven by the difference between university graduates and all others (Easterbrook et al., 2015), suggesting that this is the most important divide. Second, U.K. university entry levels are currently around 50% (http://www.bbc.co.uk/news/education-22280939), and the majority of educational policy is geared toward issues associated with access and uptake of university-level education, attesting to its wide societal and institutional significance as a status indicator. Third, a dichotomization of the education categories greatly simplifies our models, especially those that include interactions between education and identification. Finally, it maintains consistency with the experimental studies reported later.

**Table 1.** Sample Size by Highest Educational Qualification in the USS and the CS.

	USS	CS
No qualifications	4,960	2,598
GCSE	5,904	2,690
A-Level	5,404	1,775
Higher vocational	3,873	1,279
University degree	7,326	3,395
Total	27,467	11,737

*Note.* USS = Understanding Society Survey; CS = Citizenship Survey; GCSE = General Certificate of Secondary Education, exam usually taken around age 16, at the end of compulsory, full-time secondary education; A-level = qualification that marks the end of secondary education, usually taken around age 18; higher vocational = non-university post-secondary education, mainly aimed at practical and/or technical skills.

#### Identification

**Figure 1.**
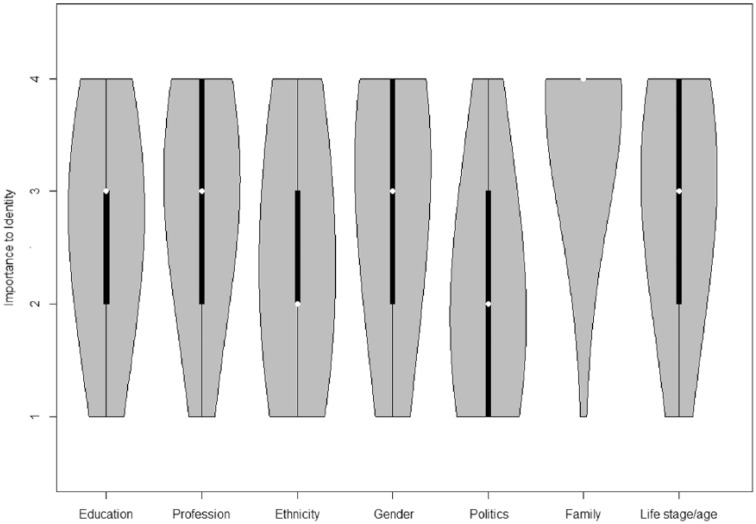
Violin plots of identification with different social categories from the Understanding Society Survey. *Note.* The white dots represent the median scores, the thick black lines represent the inner quartiles, and the thin black lines represent the outer quartiles. The gray areas show the distributions of responses.

**Figure 2.**
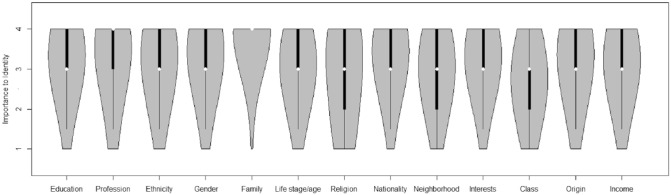
Violin plots of identification with different social categories from the Citizenship Survey. *Note.* The white dots represent the median scores, the thick black lines represent the inner quartiles, and the thin black lines represent the outer quartiles. The gray areas show the distributions of responses.

The USS and the CS data both included a question assessing the extent to which respondents incorporated different social categories into their sense of self. Respondents were asked how important social categories were “to your sense of who you are,” and the item “Your level of education” was included in this list. The other social categories included in the USS and CS are shown in Figures 1 and 2.

#### Controls

##### USS

We included several control variables in our analyses that are known to affect the outcome variables: respondents’ age and gender (1 = *male*, 2 = *female*), marital status (0 = *single*, 1 = *married or in a civil partnership*), employment status (including the categories *unemployed, employed, retired, family career, long-term illness or disability*, and *other*), and total monthly income in pounds sterling.

##### CS

We included several control variables: respondents’ age and gender (1 = *male*, 2 = *female*), marital status (0 = *single or separated*, 1 = *married*), and their income, which was coded into 15 categories.

#### Outcome variables

We recoded all analyzed continuous variables so that higher values indicate a greater endorsement of the construct.

##### USS

A self-reported health item read, “In general, would you say your health is . . .?” A life satisfaction scale (α = .81) asked how satisfied respondents were with their health, income, amount of leisure time, and their life overall. The “General Health Questionnaire 12” (GHQ12; Goldberg, 1992) was also included.

##### CS

Trust in institutions was measured by asking, “How much do you trust . . .” and then presenting three items (α = .67): “The police,” “Parliament,” and “Your local council.” A measure of life satisfaction read, “All things considered, how satisfied are you with your life as a whole nowadays?” A measure of self-reported health read, “How is your health in general?” Attitudes toward immigration were assessed by the item, “Do you think the number of immigrants coming to Britain nowadays should be increased, should be reduced, or should remain the same? Do you think that the number should be increased/reduced a little or a lot?”

### Results

#### Education and identification

We first investigated the relationship between education level and identification in both the USS and CS data. Figure 1 (USS) and Figure 2 (CS) clearly show that people incorporate their education level into their sense of who they are (USS: *M* = 2.73; CS: *M* = 3.32) and that they do this to a degree that is comparable with (and often exceeds) the extent to which they incorporate other, more frequently studied social categories such as ethnicity (USS: *M* = 2.44; CS: *M* = 3.10), gender (USS: *M* = 2.92; CS: *M* = 3.25), and nationality (CS: *M* = 3.25).

Next, we investigated the relation between people’s educational level and identification. We conducted hierarchical multiple regressions predicting identification, in which we entered the control variables in Step 1 and the degree dummy variable in Step 2. The results for the USS (Table 2) and CS (Table 3) indicate, as expected, that those with a university degree incorporated their education level into their identity to a greater extent (USS: *M* = 3.16, *SE* = .01; CS: *M* = 3.47, *SE* = .01) than did those without university degrees (USS: *M* = 2.60, *SE* = .01; CS: *M* = 3.19; *SE* = .01). The degree dummy variable accounted for an additional 6.8% of the variation in identification in the USS data and an additional 2.5% of the variation in identification in the CS data, equivalent to a medium- and small-sized effect, respectively. The CS data had higher identification means, especially for those without university degrees, suggesting that the weaker effect size associated with the degree dummy variable in the CS analyses may be due to a partial ceiling effect. Alternatively, this difference could be due to the fact that the identification question was asked during a face-to-face interview in the CS rather than self-completion questionnaire in the USS. Less educated people might be reluctant to openly admit the unimportance of their educational level in a face-to-face interview.

**Table 2.** Estimates Associated With the University Degree Dummy and Identification Variables From Hierarchical Regression Analyses of the USS Data (Study 1).

	*N*	Δ*R*^2^	*B*	*SE B*	β	*p*	95% CI	Indirect effect		
								Estimate	*SE*	95% CI
Identification	27,467									
Step 2		.068								
University degree			.563	.012	.275	<.001	[0.539, 0.588]			
Self-reported health	27,453									
Step 2		.010								
University degree			.260	.014	.106	<.001	[0.232, 0.288]			
Step 3		.001								
University degree			.236	.015	.096	<.001	[0.207, 0.264]			
Identification			.044	.007	.037	<.001	[0.030, 0.058]	.025	.004	[0.018, 0.034]
Satisfaction	27,325									
* *Step 2		.003								
University degree			.166	.018	.058	<.001	[0.132, 0.201]			
Step 3		.001								
University degree			.135	.018	.047	<.001	[0.100, 0.171]			
Identification			.055	.009	.039	<.001	[0.038, 0.072]	.031	.005	[0.021, 0.041]
GHQ12	27,184									
* *Step 2		.000								
University degree			.208	.074	.017	.005	[0.063, 0.353]			
* *Step 3		.001								
University degree			.113	.077	.009	.141	[−0.038, 0.263]			
Identification			.168	.036	.029	<.001	[0.097, 0.239]	.095	.023	[0.051, 0.137]

*Note.* USS = Understanding Society Study; CI = confidence interval; GHQ12 = General Health Questionnaire 12.

**Table 3.** Estimates Associated With the University Degree Dummy and Identification Variables From Hierarchical Regression Analyses of the CS Data (Study 1).

	*N*	Δ*R*^2^	*B*	*SE B*	β	*p*	95% CIs	Indirect effect		
								Estimate	*SE*	95% CIs
Identification	11,737									
Step 2		.025								
University degree			.286	.016	.171	<.001	[0.254, 0.318]			
Trust institutions	11,686									
* *Step 2		.007								
University degree			.122	.014	.089	<.001	[0.096, 0.149]			
* *Step 3		.003								
University degree			.110	.014	.080	<.001	[0.083, 0.137]	.013	.002	[0.008, 0.018]
Identification			.044	.008	.054	<.001	[0.029, 0.059]			
Life satisfaction	11,734									
* *Step 2		.001								
University degree			.064	.018	.036	<.001	[0.030, 0.099]			
Step 3		.005								
University degree			.042	.018	.023	.019	[0.007, 0.077]	.022	.003	[0.017, 0.029]
Identification			.078	.010	.073	<.001	[0.059, 0.098]			
Self-reported health	11,736									
Step 2		.005								
University degree			.155	.018	.080	<.001	[0.012, 0.191]			
* *Step 3		.003								
University degree			.137	.018	.071	<.001	[0.101, 0.173]	.018	.003	[0.012, 0.024]
Identification			.063	.010	.054	<.001	[0.043, 0.083]			
Immigration change attitudes	10,875									
Step 2		.029								
University degree			.415	.022	.186	<.001	[0.371, 0.458]			
* *Step 3		.001								
University degree			.402	.023	.180	<.001	[0.358, 0.447]	.012	.004	[0.005, 0.020]
Identification			.043	.013	.032	.001	[0.018, 0.067]			

*Note.* CS = Citizenship Survey; CI = confidence interval.

#### Education-based identification and health, well-being, trust, and societal attitudes

To test whether education-based identification was related to outcomes known to be affected by education, we conducted a series of hierarchical regressions. We entered the control variables into Step 1, followed by the degree dummy variable in Step 2, the identification variable in Step 3, and the identification by degree interaction in Step 4. Because all the 95% confidence intervals (CIs) associated with the interactions between identification and degree included 0, we omit the results for Step 4, but they are available from the second author on request. Finally, we used Hayes’s (2013) PROCESS macro in SPSS to conduct mediation analyses to investigate whether any of the effects of gaining a university education on our outcome variables could be accounted for by increases in identification.

The USS data show that those with a university degree reported significantly better health, were more satisfied with their life situation, and had higher mental well-being than those without a university degree (Table 2). Step 3 in the hierarchical regression indicated that, for all three outcome variables, there was a positive main effect for identification. This shows that respondents who incorporated their level of education into their identity reported better health, satisfaction with life, and mental well-being. Furthermore, none of the CIs for the indirect effects contained 0, suggesting that identification mediates some of the effect of holding a university degree on these outcomes.

The CS data indicate that, compared with those without university degrees, those with degrees reported significantly higher levels of trust in societal institutions, life satisfaction, and self-reported health, as well as more favorable attitudes toward immigration (Table 3). The results from Step 3 indicate that identification was positively related to all four outcome variables, suggesting that the more respondents incorporated their level of education into their identity, the more trust they had in societal institutions, the more satisfied they were with their lives, the better they perceived their health to be, and the more favorable their attitudes toward immigration. Finally, none of the 95% CIs associated with the indirect effects contained 0, suggesting that identification mediates some of the effect of holding a university degree on these outcomes.

### Discussion

The results from the USS and CS analyses clearly indicate that people identify with their level of education and that they do this to a degree comparable with other social categories such as ethnicity and nationality. This supports our proposition that there is merit in studying education-based identification. Also, our results are consistent with previous evidence suggesting that those with lower levels of education identify less with their level of education. Furthermore, irrespective of education level, there are psychological benefits to be gained from incorporating education level into one’s identity: The more respondents did so, the higher were their self-reports of health, satisfaction with life, mental well-being, favorable attitudes toward immigrants, and levels of trust in societal institutions. Finally, increases in identification accounted for some of the beneficial effects of having a university degree.

Although these initial results are encouraging, the single-item measures of identification included in the USS and CS are relatively crude and prohibit conclusions about which facets of identification are driving these effects. Social identification is known to consist of several facets (e.g., Leach et al., 2008), and it is possible that the different aspects have distinct effects. For example, given that education is perceived to be the result of personal achievement, *affective identification*, that is, satisfaction with one’s educational group membership, could play a special role. Less educated people might struggle to find satisfaction in their relatively low level of education, and this is likely to affect self-esteem. Indeed, manipulations that devalue one’s in-group have been shown to most strongly affect the satisfaction aspect of identification (Leach, Mosquera, Vliek, & Hirt, 2010). Affective identification thus reflects the *value* that is attached to group belonging, whereas other identification aspects reflect a more traditional view of identification: the importance of the group to the individual.

There is evidence that different identification aspects play different roles for the unemployed, a group that has a similar societal stigma attached to it as the less educated. Affective identification with being unemployed (e.g., “I feel good in the group of unemployed people”) is positively related to self-esteem and perceived health, whereas a more cognitive identification dimension (e.g., “I identify with unemployed people”) shows a negative relation with self-esteem and perceived health (Herman, Bourguignon, Stinglhamber, & Jourdan, 2007). It may be, therefore, that dimensions of identification that focus on the affective satisfaction that is derived from group memberships are universally beneficial for self-esteem, well-being, and social attitudes. However, identification without this satisfaction element reflects a more cognitive awareness of the group and its importance to one’s identity. It may be that this non-affect identification could have negative consequences for members of stigmatized groups as their low status becomes internalized (Crabtree et al., 2010).

We therefore conducted two further studies to investigate which aspects of identification are central to the effects found in Study 1. Study 2 investigates the relation between education and identification in a U.K. sample; Study 3 does the same with a U.S. sample and, in addition, investigates the relation between different facets of identification and a wide range of outcome variables known to be related to education.

## Study 2

In Study 2, we investigated whether identification with one’s educational group is different for people with or without a university degree, but we used a multidimensional measure of identification rather than the single-item measures available in Study 1.

### Method

#### Participants

Initially, 208 participants were recruited through a research assistant’s social network (this was the maximum that could be achieved within the available time frame). Thirty-seven participants who did not provide information about their educational level or did not answer the identification questions were excluded from analyses. Three participants who were 15 or 16 years old and still in secondary education were also excluded; 168 remained (age *M* = 24.5, *SD* = 5.7; 65 male, 97 female, 6 gender unknown). In view of the promising results (the effect of education on identification already being significant) and the relatively small sample, we decided to recruit additional participants. A further 314 participants were recruited through an online loyalty program (www.maximiles.co.uk); by way of compensation, they received points that could be exchanged for consumer purchases. Forty participants who did not provide information about their educational level or did not answer the identification questions were excluded from analyses. One participant was excluded because he responded “1” to 42 consecutive questions; 273 participants remained. Thus, in total, there were 441 participants (293 female, 129 male, 19 gender unknown; age *M* = 32.78, *SD* = 11.50). Participants completed an online questionnaire.

#### Education

Participants were asked to indicate the highest educational level they had achieved, and responses were again recoded into two categories: No university degree (*n* = 210) and University degree (*n* = 231). Because we had a young sample, and 19.3% were still in full-time education, we categorized those who were currently students as holding the degree or certificate for which they were studying.

#### Identification

Identification was assessed immediately after the question about participants’ level of education. We used 10 items from Leach et al.’s (2008) multidimensional identification scale, 2 items for each subscale (e.g., “I feel a bond with people who have had the same education as me”). Scores for solidarity (*r* = .79), satisfaction (*r* = .85), centrality (*r* = .87), self-stereotyping (*r* = .63), and homogeneity (*r* = .80) were computed by averaging the items.

### Results and Discussion

As expected, in a multiple regression analysis controlling for gender and age and with education represented as a dummy variable, identification with one’s educational group was higher among those with a university degree (see Table 4). The strength of the relation between education and identification differed substantially between the identification dimensions; the change in *R*^2^ due to the education variable varied from .004 to .124. The largest effect size was for satisfaction, followed by centrality; the smallest was for in-group homogeneity. Satisfaction is an affective dimension of identification that is closely related to private collective self-esteem (Luhtanen & Crocker, 1992) and measures how happy and satisfied people are to have a particular level of education. In other words, lower educated people do not feel good about their level of education. We interpret this as evidence that low levels of education form a basis for social stigma and status. In Study 3, we investigate whether this stigma is related to a series of outcomes central to the education effect.

**Table 4.** Relation Between Education and Identification With Education Group (Study 2).

	No university degree	University degree	95% CI for difference in means	*R*^2^ change
Solidarity	3.94	4.40	[0.17, 0.74]	.022**
Satisfaction	4.91	5.95	[0.78, 1.29]	.124***
Centrality	4.30	5.30	[0.71, 1.29]	.094***
Individual self-stereotyping	3.89	4.44	[0.28, 0.80]	.036***
In-group homogeneity	3.77	3.95	[−0.10, 0.47]	.004

*Note.* CI = confidence interval.

* *p* < .05. **p < .01. ***p < .001.

## Study 3

We found that less educated people identify much less with their educational level than higher educated people do, especially for an affective dimension of identification. We now investigate how education-based identification relates to a series of outcomes central to the education effect. Study 3 is therefore similar to Study 1, but now we used a multidimensional measure of identification.

We also included a manipulation of the *salience of people’s educational level* because we wanted to investigate experimentally the effects of a contextual cue for the importance of education. People are often confronted with reminders of their education level, such as completing forms that ask about educational qualifications. Educational salience is expected to have two related effects. First, it could strengthen the relation between identification and the outcome variables, simply because making education salient focuses respondents’ attention on this aspect of their identity. Second, the education salience manipulation could have direct, experimental effects on the outcome variables. Such effects should corroborate the relations between identification and the outcome variables and are a complementary way of investigating the importance of education-based social identity. Identification and education salience are person-related and situation-related indicators of the importance of social identity, respectively. Both personal and situational factors are known to play a role in social identity effects, and they frequently interact with each other, with situational importance often amplifying the effect of personal importance (see Spears, Doosje, & Ellemers, 1999). For example, if high education-based identification is related to greater well-being, it is to be expected that making education salient will increase well-being, especially for those who identify highly with their educational group. Finding effects of both identification and education salience would therefore provide strong support for the role of education-based identity in well-being and societal attitudes.

We measured participants’ educational level and their identification with their educational group as predictor variables and manipulated the salience of people’s educational level. As dependent variables, we included a wide range of variables known to be related to education. Having no basis to estimate effect sizes, we initially recruited a sample of about the same size as Study 2. Several of the three-way interactions reported below were already significant, so we ran another, equally large study to obtain more robust results. For analytic purposes, we pooled the data from these two studies (Studies 3a and 3b). Unless indicated otherwise, all questions were identical across the two studies.

### Analytic Strategy and Overview

We first report the education main effects (not controlling for identification) to test whether well-established education effects are replicated. Then, we address the issue of identification and how best to conceptualize and operationalize this. Specifically, we argue that making a distinction between esteem-related and non-esteem-related identification sheds important light on the role of identification. In Study 2, we already found that the effect of education was strongest for esteem-related aspects of identification.

Our main analyses focus on (a) how identification (both group esteem and non-esteem identification) related to the outcome variables and (b) whether the outcome variables were affected by the education salience manipulation. We therefore investigate both person-related (identification) and situation-related (education salience) indicators of the importance of social identity, and this is the main purpose of Study 3.

### Method

In Study 3a, 420 MTurk workers (157 female, *M*_age_ = 30.7, *SD*_age_ = 11.1) completed an online questionnaire. Nineteen participants did not answer “agree strongly” to the question “Please select the ‘agree strongly’ answer,” and a further 18 did not disagree with the item “I am an elephant and I live in Africa.” These 37 inattentive participants were excluded from all analyses (383 remained).

In Study 3b, 532 MTurk workers (340 female, *M*_age_ = 34.7, *SD*_age_ = 12.4) completed an online questionnaire. Forty participants failed the same attention check items as those used in Study 3a and were excluded from all analyses (492 remained).

#### Salience of education

Participants were randomly assigned to the “Education salient” or the “Education not salient” condition. Questions about the education of respondents were answered either before (Education salient) or after (Education not salient) the dependent variables. The education questions were about respondents’ highest educational level and the field of their highest qualification, and also included all identification items and the “importance of education” item used in Study 1. We assume that education would have been salient for participants who had answered the education questions before they responded to the dependent variables.

#### Education

Participants’ highest educational level was recoded into two categories: no 4-year college degree (*n* = 422) and at least a 4-year college degree (*n* = 453).

#### Identification

We used the same multidimensional identification scale as used in Study 2 (Leach et al., 2008) but now included all 14 items. We added 3 items intended to measure public collective self-esteem (e.g., “In general, others respect people with my level of education,” adapted from Luhtanen & Crocker, 1992). Scores for solidarity (α = .89), satisfaction (α = .92), centrality (α = .81), self-stereotyping (*r* = .70), homogeneity (*r* = .72), and public collective self-esteem (α = .82) were computed by averaging responses to the relevant items.

#### Outcome variables

Rather than focus on one particular variable, we included constructs reflecting the wide range of education effects found in the literature. In addition to life satisfaction, we included measures of social attitudes such as political attitudes and attitudes toward minorities. We used formulations and response options identical to those in representative surveys to be able to relate the results of Study 3 to the education effects found in previous research (including Study 1).

##### Life satisfaction

We asked participants, “In general, how satisfied would you say you are with your life?”

##### Political attitudes

To measure *interest in politics*, we asked, “How much interest do you generally have in what is going on in politics?” Higher scores reflected greater interest in politics. *Political cynicism* was measured with two items (*r* = .34), for example, “Generally speaking those we elect as members of Congress lose touch with people pretty quickly.”

##### Intergroup attitudes

Three items (α = .83) measured *negative attitudes toward immigrants*, for example, “Immigrants are generally good for the U.S. economy” (reverse-scored). Two items (*r* = .62) measured *symbolic racism* (Henry & Sears, 2002), for example, “Irish, Italians, Jews, and many Other minorities overcame prejudice and worked their way up. Blacks should do the same without any special favors.”

### Results

Means, standard deviations, and correlations for all variables are shown in Table 5. As explained above, we first report the education main effects. Then, we investigate the operationalization of identification and its relation with education. Finally, our main analyses concern the effects of identification and education salience on the outcome variables (i.e., well-being and social attitudes).

**Table 5.** Descriptive Statistics for Study 3.

	*N*	*M*	*SD*	Scale endpoints	1	2	3	4	5	6
1. Non-esteem identification	875	0.236	1.195	−3, 3						
2. Group esteem	875	0.702	1.342	−3, 3	.64***					
3. Life satisfaction	874	3.180	1.100	1, 5	.25***	.35***				
4. Interest in politics	875	3.274	1.028	1, 5	.07*	.08*	.08*			
5. Political cynicism	875	1.412	1.179	−3, 3	−.09*	−.08*	−.07*	.03		
6. Negative attitudes toward immigrants	875	−0.537	1.429	−3, 3	−.12***	−.14***	−.04	−.10**	.05	
7. Symbolic racism	875	−0.091	1.594	−3, 3	−.06	−.08*	.05	−.07*	.10**	.53***

* *p* < .05. **p < .01. ***p < .001.

#### Education main effects

As expected, higher education (controlling for age, gender, and education salience, but not for identification) was related to marginally greater life satisfaction (*B* = .14, *SE* = .074, *p* = .06, 95% CI [0.273, 0.421]), greater interest in politics (*B* = .18, *SE* = .068, *p* = .009, 95% CI [0.044, 0.311]), less negative attitudes toward immigrants (*B* = −.48, *SE* = .124, *p* < .001, 95% CI [−0.727, −0.241]), and less symbolic racism (95% CI [0.273, 0.421], *B* = −.45, *SE* = .142, *p* = .002), compared with those without a 4-year college degree. The effect of education on political cynicism was in the expected direction but not significant (*B* = −.11, *SE* = .080, *p* = .16, 95% CI [−0.726, −0.168]).

#### Identification

Identification with one’s educational group was again higher among the more highly educated (see Table 6). Consistent with the findings of Study 2, the largest effects were for public collective self-esteem and satisfaction. It is clear that facets of identification related to group esteem show the strongest relation with education. In other words, the lower educated do not find it pleasant to belong to their group and do not think that others have a favorable opinion of it. The two esteem-related dimensions, satisfaction and public collective self-esteem, also correlate strongly (*r* = .76). For the analyses of the relations between identification and well-being/social attitudes, we therefore separated esteem-related from non-esteem-related aspects of identification. Satisfaction and public collective self-esteem items were combined into a single scale of *group esteem* (α = .92). The remaining identification dimensions were also highly related (*r*s between .40 and .71). To prevent multicollinearity, we combined the solidarity, centrality, individual self-stereotyping, and in-group homogeneity items into a measure of *non-esteem identification* (α = .91). This measures how subjectively important the educational group is, without reference to the hedonic value of the group.

**Table 6.** Relation Between Education and Identification With Education Group (Study 3).

	No 4-year college degree	4-year college degree	95% CI for difference in means	*R*^2^ change
Solidarity	−.25	.54	[0.61, 0.98]	.075**
Centrality	−.05	.90	[0.76, 1.14]	.098***
Individual self-stereotyping	.04	.44	[0.22, 0.58]	.022***
In-group homogeneity	−.19	.23	[0.24, 0.61]	.023***
Satisfaction	.09	1.40	[1.14, 1.48]	.215***
Public collective self-esteem	−.18	1.34	[1.36, 1.69]	.274***

*Note.* CI = confidence interval.

* *p* < .05. **p < .01. ***p < .001.

#### Effects of identification and salience of education

We use multiple regression models including age, gender, educational level, salience of education, and group esteem and non-esteem identification as predictors. We also included all two-way and three-way interactions between education, salience, and group esteem and non-esteem identification, but no interactions including both group esteem and non-esteem identification at the same time.

##### Life satisfaction

**Figure 3.**
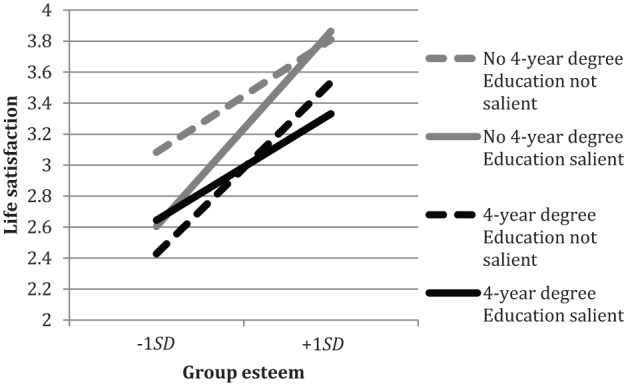
Group esteem and life satisfaction, by participant education and education salience. *Note.* Group esteem consists of satisfaction and public collective self-esteem.

There was a main effect of group esteem, *B* = .35, *SE* = .038, *p* < .001, 95% CI [0.273, 0.421], showing that, overall, higher group esteem was related to higher life satisfaction. It is worth noting that the education main effect changed from positive to negative, *B* = −.36, *SE* = .081, *p* < .001, 95% CI [−0.520, −0.202], when group esteem was added to the model (see Figure 3); we return to this point in the “Discussion” section. The main effect for non-esteem identification was not significant, *B* = .03, *SE* = .038, *p* = .46, 95% CI [−0.047, 0.103], suggesting that the effect found in Study 1 might have been due mainly to esteem-related aspects of identification.

There were also three-way interactions between education level, education salience, and both group esteem and non-esteem identification (*B* = −.36, *SE* = .16, *p* = .03, 95% CI [−0.669, −0.041] and *B* = .35, *SE* = .15, *p* = .02, 95% CI [0.046, 0.646], respectively). We discuss these in turn, starting with *group esteem*. As can be seen in Figure 3, all relations between group esteem and life satisfaction were positive. When education had been made salient, the relation between group esteem and life satisfaction was stronger for those without a 4-year college degree, *B* = .47, *SE* = .067, *p* < .001, 95% CI [0.338, 0.604], than for those with such a degree, *B* = .28, *SE* = .101, *p* = .007, 95% CI [0.078, 0.477]. Thus, group esteem is beneficial for the life satisfaction of all respondents but is especially beneficial for those with lower levels of education.

Turning to the effects of educational salience, we decomposed the three-way interaction in a different way to examine the simple effects of education salience. Only one simple effect was significant. This showed that the lower educated who reported low levels of group esteem had significantly lower life satisfaction when education had been made salient (*M* = 2.32) compared with when it had not been made salient (*M* = 2.88), 95% CI for difference [0.246, 0.866], *F*(1, 862) = 12.39, *p* < .001, $\eta_{p}^{2}$ = .014.^1^ In other words, the lower educated who are low in group esteem reported lower life satisfaction after their own educational level had been made salient.

**Figure 4.**
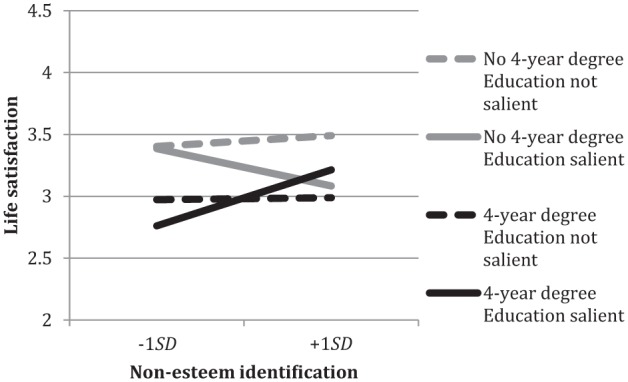
Non-esteem identification and life satisfaction by participant education and education salience. *Note.* Non-esteem identification consists of centrality, solidarity, individual self-stereotyping, and in-group homogeneity.

Turning to the three-way interaction with *non-esteem identification*, Figure 4 shows that when education had been made salient, non-esteem identification positively predicted life satisfaction for those with a 4-year degree, *B* = .21, *SE* = .086, *p* = .02, 95% CI [0.036, 0.376], but negatively for those without, *B* = −.12, *SE* = .072, *p* = .10, 95% CI [−0.260, 0.022]. Although the latter relation is not statistically significant, it contrasts markedly with the positive relation observed for the higher educated.

We again decomposed the interaction to examine the simple effects of education salience. This confirmed the negative role of non-esteem identification for the less educated. Indeed, less educated respondents high in non-esteem identification reported lower life satisfaction when education had been made salient (*M* = 2.78) compared with when it had not been made salient (*M* = 3.27), 95% CI for difference [0.184, 0.798], *F*(1, 862) = 9.84, *p* = .002, $\eta_{p}^{2}$ = .011. No other simple effects of education salience were significant.

In summary, when education had been made salient, both low group esteem and high non-esteem identification were associated with lower life satisfaction for participants without a 4-year college degree, compared with those with a 4-year degree.

##### Interest in politics and political cynicism

**Figure 5.**
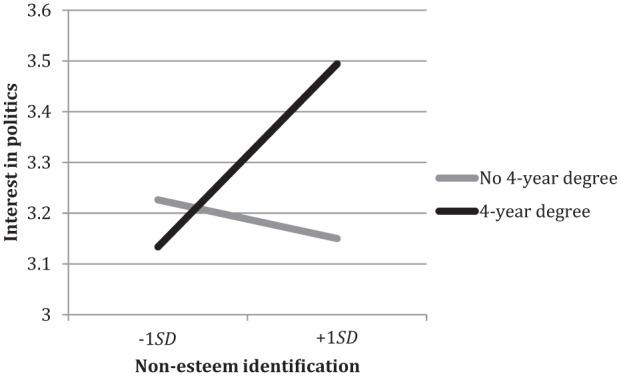
Non-esteem identification and interest in politics by participant education. *Note.* Non-esteem identification consists of centrality, solidarity, individual self-stereotyping, and in-group homogeneity.

There were no significant main effects of group esteem, *B* = .01, *SE* = .037, *p* = .88, 95% CI [−0.068, 0.078], or non-esteem identification, *B* = .06, *SE* = .038, *p* = .10, 95% CI [−0.012, 0.135]. Group esteem was not involved in any significant interaction effects. However, non-esteem identification interacted with education, *B* = .18, *SE* = .08, *p* = .02, 95% CI [0.034, 0.331]: Figure 5 shows that non-esteem identification was related to greater interest in politics among people with a 4-year college degree, *B* = .14, *SE* = .057, *p* = .02, 95% CI [0.025, 0.250], but not among those without a degree, *B* = −.03, *SE* = .050, *p* = .61, 95% CI [−0.124, 0.073]. No other effects of identification were significant. There were no interactions between identification and education salience, so we did not examine education salience simple effects. Results for *political cynicism* were very similar to those for interest in politics. For the sake of brevity, they are not reported here, but details can be found in the online supplemental materials.

In summary, higher non-esteem identification was related to more positive attitudes toward politics for the higher educated but not for the less educated.

##### Negative attitudes toward immigrants

For these analyses, we selected participants who self-identified as European Americans, were born in the United States, and whose parents were both born in the United States (*N* = 553). There were no main effects of group esteem, *B* = −.08, *SE* = .067, *p* = .22, 95% CI [−0.213, 0.048], or non-esteem identification, *B* = −.03, *SE* = .069, *p* = .68, 95% CI [−0.164, 0.107]. Group esteem interacted with education salience, *B* = .27, *SE* = .134, *p* = .04, 95% CI [0.009, 0.536]. Unexpectedly, group esteem was related to less negative attitudes toward immigrants when education had not been made salient, *B* = −.20, *SE* = .093, *p* = .03, 95% CI [−0.384, −0.017], but not when education had been made salient, *B* = .07, *SE* = .095, *p* = .47, 95% CI [−0.119, 0.256].

**Figure 6.**
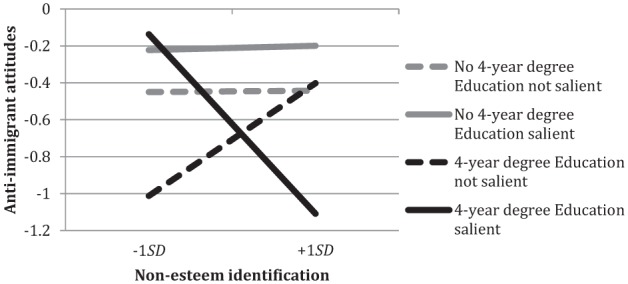
Non-esteem identification and anti-immigrant attitudes by participant education and education salience. *Note.* Non-esteem identification consists of centrality, solidarity, individual self-stereotyping, and in-group homogeneity.

More interestingly, there was a three-way interaction between non-esteem identification, education, and education salience, *B* = −.68, *SE* = .279, *p* = .01, 95% CI [−1.232, −0.136]. Regarding the relation between non-esteem identification and negative attitudes toward immigrants, Figure 6 shows that when education was salient, non-esteem identification was related to less negative attitudes toward immigrants for those with a 4-year college degree, *B* = −.41, *SE* = .139, *p* = .004, 95% CI [−0.687, −0.138], but not for those without a degree, *B* = .01, *SE* = .125, *p* = .92, 95% CI [−0.235, 0.260].^2^ Thus, higher non-esteem identification played a more positive role (assuming that positive attitudes to immigrants are desirable) for those with a college degree than for those without a degree.

When we decomposed this interaction to examine the simple effects of education salience, we again found evidence of the positive role of non-esteem identification for the higher educated. Among the higher educated participants, those for whom education had been made salient (compared to those for whom education had not been made salient) reported attitudes toward immigrants that were less negative when they were high in non-esteem identification (*M* = −1.08 vs. −0.43, 95% CI for difference [0.090, 1.228]), *F*(1, 541) = 5.18, *p* = .02, $\eta_{p}^{2}$ = .009, but more negative when they were low in non-esteem identification (*M* = −0.17 vs. −.87, 95% CI for difference [0.127, 1.261]), *F*(1, 541) = 5.78, *p* = .02, $\eta_{p}^{2}$ = .011.

In sum, both the correlational and the experimental evidence point to the same conclusion: Higher non-esteem identification leads the higher educated, but not the less educated, to report less negative attitudes toward immigrants.

##### Symbolic racism

For these analyses, we again selected the subset of 553 participants who self-identified as European Americans, were born in the United States, and whose parents were both born in the United States. There were no main effects of group esteem, *B* = .004, *SE* = .077, *p* = .96, 95% CI [−0.146, 0.155], or non-esteem identification, *B* = .02, *SE* = .079, *p* = .78, 95% CI [−0.133, 0.178].

Group esteem also did not have any interaction effects. However, there was a three-way interaction between non-esteem identification, education, and education salience, *B* = −.76, *SE* = .324, *p* = .02, 95% CI [−0.401, −0.128] (see Figure S2 in the online supplemental material). None of the simple effects of non-esteem identification were significant (all *p*s > .13), but when we decomposed this interaction by salience condition, the two-way interaction between education and non-esteem identification was marginally significant when education was salient (*B* = −.42, *SE* = .217, *p* = .054, 95% CI [−0.846, 0.007]) but not when it was not salient (*B* = .36, *SE* = .243, *p* = .14, 95% CI [−0.115, 0.841]). This reflects the fact that when education had been made salient, higher non-esteem identification was associated with less symbolic racism among the higher educated, *B* = −.21, *SE* = .164, *p* = .20, 95% CI [−0.538, 0.111], and with more symbolic racism among the less educated, *B* = .17, *SE* = .144, *p* = .23, 95% CI [−0.112, 0.459], although the simple slopes were non-significant.

Decomposing the interaction differently to examine the simple effects of education salience, there was a corresponding effect of education salience: For the higher educated who reported high levels of non-esteem identification, making education salient resulted in marginally less racism (*M* = −.15) than when education had not been made salient (*M* = .45), 95% CI for difference [−0.055, 1.270], *F*(1, 541) = 3.25, *p* = .07, $\eta_{p}^{2}$ = .006.

Although these effects on symbolic racism were less strong than those for other outcome variables, the pattern is clearly similar: Non-esteem identification was more strongly related to harmonious social attitudes for those with a college degree than for those without one, especially when education had been made salient.

### Discussion

*Non-esteem identification* had more positive consequences for the well-being and social attitudes of higher educated people than for those of lower educated people. This was the case for interest in politics and political cynicism, and when education had been made salient, it was also true for life satisfaction, anti-immigrant attitudes, and symbolic racism. Importantly, these correlational findings were echoed by the experimental effects of making education salient. For the less educated who were high in non-esteem identification, making education salient led to lower life satisfaction. In contrast, for the *higher* educated who were high in non-esteem identification, education salience led to less symbolic racism and less negative attitudes toward immigrants. Thus, education-based identity only had positive effects for the higher educated.

This is an important finding because it shows that identification, when analyzed separately from esteem-related aspects of identification, is a not a positive factor for people with lower levels of formal education. The identity content of having lower levels of education is sufficiently negative that incorporating it into the self-concept does not have any beneficial effect. This runs counter to established findings regarding the beneficial effects of group membership and identification (Crabtree et al., 2010; Jetten et al., 2012; Schmitt & Branscombe, 2002). However, our results are in line with work showing negative effects of identification with being unemployed (Herman et al., 2007), which implies that there are inherently negative consequences of defining the self on a consensually devalued dimension.

In contrast to non-esteem identification, the positive relation between *group esteem* and life satisfaction was stronger for people with lower rather than higher levels of education when education had been made salient. High group esteem means that people value the group highly and think that others do so, too, and this seems to foster a rejection of the negativity of being less educated. Less educated people who are high in group esteem apparently have sufficient resources to challenge the negative identity of being less educated. Also, both group esteem and life satisfaction reflect positivity toward the self, further explaining their relation. This interpretation is consistent with the fact that people who identify with the less educated perceive the less educated to be warmer *and* more competent than the higher educated (Spruyt & Kuppens, 2014). This appears to be an example of the classic way in which identification buffers against negative social identity (Schmitt & Branscombe, 2002), although in the present case, this only occurred for esteem-related identification. Indeed, the less educated had higher life satisfaction than the higher educated, once group esteem was controlled for. It is important to add, however, that this coping mechanism of increasing group esteem is not available to many in the lower educated group, because they have much lower group esteem than do the more highly educated in the first place. In Study 3, for example, 70% of those with a 4-year college degree scored above the sample median for group esteem, whereas only 25% of those without a 4-year college degree did so. Thus, in addition to the lack of a positive role of non-esteem identification for the less educated, the beneficial effects of group esteem are likely to be limited.

In contrast to Study 1, we found that the effect of identification differed between people with or without a university degree. This difference between Studies 1 and 3 seems to be due to the identification measure used. The one used in Study 3 was multi-item and multidimensional. Consistent with this argument, when we analyzed the Study 3 data using the same identification measure as in Study 1 (which had also been included in Study 3), the results were consistent with those found in Study 1 (details are available in the online supplemental materials), thereby arguing against an alternative explanation that differences in the findings of Studies 1 and 3 reflect differences in the nationality or representativeness of the samples.

## General Discussion

We investigated education-based social identity and how it is related to well-being and social attitudes. Overall, our results suggest that education-based social identity plays a role in a wide range of outcomes associated with low education, such as low well-being and political and intergroup attitudes that indicate threats to social cohesion.

We found strong support for the hypothesis that education-based identification is lower among those with lower levels of education. This was the case in all three studies, one of which was based on representative samples of the population. Moreover, the relation between education and identification was especially strong for aspects of identification related to group esteem, possibly reflecting social reality constraints: It is easier to be positive about higher qualifications. In addition to the obviously negative connotations of having a lack of education, educational attainment is frequently regarded as a legitimate reflection of individual merit. Given these constraints, it is difficult for the less educated to have high group esteem. Theoretically, esteem-related aspects of identification have sometimes been conceptualized as distinct from identification. For example, Correll and Park (2005) called this the perceived value of the group, a factor they separate from identification.

Study 3 confirmed that group esteem and non-esteem identification had different relations with outcome variables, especially when education was made salient. Unlike non-esteem identification, group esteem had a positive relation with life satisfaction for the less educated. This shows that resisting the negative aspects of their education-based identity pays off for the less educated. However, the corollary is that low group esteem had negative effects, especially when education was salient. It should also be borne in mind that in absolute terms, group esteem was generally low among the less educated, meaning that its beneficial effects are likely to be limited. Setting aside these esteem-related aspects, identification itself was related to higher life satisfaction and more positive political and intergroup attitudes for the more highly educated but not for the less educated.

The fact that the results for education-based identity were consistent for a wide range of outcomes relating to well-being and social attitudes is an important strength of our research. A common underlying explanation opens up the prospect of reducing these diverse negative effects through a single intervention. At the same time, it is worth acknowledging that each outcome variable is likely to have a particular relation with education and that the pathways from education to these outcomes may vary to some extent from one outcome to another. For example, the exact mediators could differ between the outcome variables. Education-based identification, nevertheless, offers a good starting point for investigating these relations.

Do people really identify with education-based groups, and are their responses to the identification items meaningful? These are important questions because education-based identification has rarely been studied. Several pieces of evidence from the current research suggest that an education-based identity *is* a meaningful concept. In Study 1, respondents indicated that their level of education was as important to their sense of who they are as their gender or nationality and more important than their ethnicity. Furthermore, endorsement of stereotypes about education-based groups depends to some extent on group identification (Spruyt & Kuppens, 2014). All this evidence supports the idea that people’s level of education can be a basis for social identity.

The current work advances knowledge in several ways. First, it qualifies previous research showing the beneficial effects of identification. For some groups, identification—at least when its esteem-related aspects have been removed—does not have a beneficial effect. Similar evidence has been reported in the case of the unemployed (Herman et al., 2007). Future research should clarify whether esteem-related and non-esteem-related aspects of identification also play different roles in other disadvantaged groups and the conditions under which this occurs. Second, we have studied a group with a very particular negative social identity that may be difficult to cope with because it is associated with high personal responsibility. In this respect, low education is similar to obesity (Klaczynski, Goold, & Mudry, 2004). Having a low level of education is seen not only as inherently negative but also as a legitimate reflection of individual merit, thereby rendering traditional coping strategies problematic.

Future research on education as a source of social identity could build on our conclusion that the negative effects of being less educated result from holding a stigmatized identity by using self-affirmation manipulations to attenuate these education effects (Harackiewicz et al., 2014). Other research could investigate how the less educated view their level of education and their position in society. Their identification is low, but how do they explain and deal with this low status? A research question that has been discussed in sociology is whether the education divide has the potential to be a basis of societal conflict (see Spruyt & Kuppens, 2014). Should we expect collective action on the part of the less educated, and if so, in what form? Or does their lack of identification imply acceptance and even self-exclusion? Given the current importance of education as a structural factor in society, such research is surely timely.

In conclusion, a key message of our research is that despite having been neglected as a research topic in psychology, education provides a basis for a social identity. Our research shows that education-based identity is associated with positive outcomes, especially for the higher educated. However, the merit-based ideology infusing this particular identity undermines these positive effects for the less educated, with clear negative consequences, both for the individual and for society.

## Notes

1. All simple effects reported here were calculated at 1 *SD* above or below the mean of respondents with or without a 4-year college degree, rather than 1 *SD* above or below the sample mean.

2. The simple effect of non-esteem identification for the higher educated was also not significant when education was not salient, *B* = .22, *p* = .18.
